# Retrospective Ratings of Emotions: the Effects of Age, Daily Tiredness, and Personality

**DOI:** 10.3389/fpsyg.2015.02020

**Published:** 2016-01-11

**Authors:** Aire Mill, Anu Realo, Jüri Allik

**Affiliations:** ^1^Department of Psychology, University of TartuTartu, Estonia; ^2^Estonian Academy of SciencesTallinn, Estonia

**Keywords:** retrospective ratings, emotional memory, age, daily tiredness, personality

## Abstract

Remembering the emotions we have experienced in the past is the core of one's unique life-experience. However, there are many factors, both at the state and trait level that can affect the way past feelings are seen. The main aim of the current study was to examine the impact of individual differences on systematic biases in retrospective ratings compared to the momentary experience of basic emotions such as sadness, fear, happiness, and anger. To this end, an experience sampling study across 2 weeks was conducted using a younger and an older age-group; the experience of momentary emotions was assessed on 7 randomly determined occasions per day, the retrospective ratings being collected at the end of each day about that day, as well as at the end of the study about the previous 2 weeks. The results indicated that age and daily tiredness have significant effects on retrospective emotion ratings over a 1-day period (state level), enhancing the retrospective ratings of negative emotions and decreasing the ratings of felt happiness. Whereas personality traits influence the more long-term emotion experience (trait level), with all Big Five personality traits having selective impact on retrospective emotion ratings of fear, sadness, happiness, and anger. Findings provide further evidence about the systematic biases in retrospective emotion ratings, suggesting that, although retrospective ratings are based on momentary experience, daily tiredness and personality traits systematically influence the way in which past feelings are seen.

## Introduction

Remembering one's emotions over time is at the core of human life experience, and we all have emotional memories that are vivid and lasting. Recent research suggests that people remember their emotions quite accurately both after 90 days (Barrett, 1997) and after 1 year (Röcke et al., 2011). However, retrospective ratings of experienced affect are also susceptible to systematic biases. It has been found that individuals retrospectively overestimate both positive and negative affect when comparing momentary reports with end-of-day ratings (Thomas and Diener, 1990; Parkinson et al., 1995). Age, among other individual difference factors, has also been found to influence both the momentary and retrospective ratings of emotions (Röcke et al., 2011). In addition to individual differences and the respondent's current mood, memory about emotional experiences can also reflect the emotion regulation strategies used in a particular situation. Emotion regulation refers to attempts to influence one's subjective emotion experience and expression, involving both up- and down-regulation, as well as antecedent-focused and response-focused emotion regulation (Gross et al., 2006). Emotion regulation strategies are thought to differ across age-groups (Urry and Gross, 2010), and across emotion categories (Gross et al., 2006). A recent study by Shallcross et al. (2012) showed that the nature of age-related decreases in emotional processing is best understood across discrete emotions. The current study extends research on retrospective emotion by comparing retrospective and momentary emotion reports. Further, the impact of daily tiredness and personality on such reports are explored.

### Momentary vs. retrospective reports

Current study used experience sampling methodology to capture ratings of emotional experiences as dynamic psychological processes. The advantages of experience sampling compared to nomothetic approaches, is the focus on individuals' current or very recent experiences and the usage of multiple assessments over time in everyday life (Trull and Ebner-Priemer, 2009). Retrospective self-reports may be biased by variety of heuristics, the retrieved emotional states are summarized by taking also into account the personal relevance and significance of reported experiences, as well as social expectations (e.g., Wilhelm and Grossman, 2010). However, repetitive self-ratings and frequent monitoring may produce other biases, for example response shifts (Schwartz et al., 2006). It has been found that individuals retrospectively overestimate both positive and negative affect when comparing momentary reports with retrospective ratings (Thomas and Diener, 1990; Parkinson et al., 1995). The Accessibility Model of Emotional Self-Report by Robinson and Clore (2002) proposes different levels of remembering based on accessibility principles, and makes the distinction between momentary emotions, short-term retrospective reports (i.e., end of day), and longer-term reports (i.e., a week or multiple weeks), with each of these judgments being influenced by different sources of knowledge. Short-term judgments are influenced by more episodic forms of memory biases or episodic knowledge such as salient experiences and current affective state. By contrast, longer-term retrospective reports are influenced by more semantic forms of knowledge, including the beliefs and theories about self, and one's personality. The age, neuroticism, extraversion, and tiredness can be expected to influence retrospective emotion ratings, but there is no clear timeline distinction provided by previous studies (Carstensen et al., 2011; Röcke et al., 2011).

According to accessibility model of emotional self-reports, the current emotions are accessed directly based on experiential knowledge, specific moments from the past can be retrieved from the episodic memory, but also from the semantic knowledge—the situation-specific beliefs and identity-related beliefs (Robinson and Clore, 2002). The amount of time between the event and the recall defines the extent of accessible episodic memories, and the shift from episodic to semantic memory, i.e., situation-specific and identity-based beliefs (Tulving, 1984; Robinson and Clore, 2002). According to the two-process model, the recollection of emotion experience over the longer time-frame (i.e., *last few weeks*) has found to rely on semantic memories, whereas for shorter periods (i.e., *past day)* the episodic knowledge is used (Robinson and Barrett, 2009). There is evidence that contrary to peak-end rule in memories for pain experiences (Redelmeier et al., 2003), the retrospective evaluations of multiepisode emotion experience across 1 day rely rather on averaged ratings of emotions (Miron-Shatz, 2009; Röcke et al., 2011).

There are distinct types of memory biases in retrospections due to episodic (factors affecting retrieval of event-related context) vs. semantic knowledge or beliefs about emotions (Robinson and Clore, 2002). Personality can be viewed as a source of beliefs that contribute differentially to online and retrospective emotion reports, with being related rather to semantic memory (Robinson and Clore, 2002). Age is another factor that can be expected to interfere with both online and retrospective emotion reports, the question is whether more as a short-term (retrieving the context of event) or long-term (regarding event-specific or general beliefs about the influence of age on emotion experience). Thirdly, the everyday tiredness can be expected to act more as an episodic biasing factor inferring the recollection of contextual details of experience as tiredness itself is rather situational and contextual feature.

### Age and emotional memory

Previous studies have suggested that age differences in emotional long-term memory reflect a memory retrieval bias, meaning that the emotional life of older people is different, as emotional memories have a different meaning for them (Spaniol et al., 2008). It has also shown that older people tend to experience specific mood states (e.g., nocturnal regrets) that are rare in younger adults (e.g., Schmidt et al., 2011).

According to socioemotional selectivity theory, older adults are expected to endorse more positive stimuli during memory retrieval than younger adults (Carstensen et al., 2011). Urry and Gross (2010) proposed a SOC-ER (selection, optimization, and compensation with emotion regulation) framework of emotion regulation processes that facilitate the emotional positivity effect. According to this framework, older adults use more effective situation selection by reducing the probability of engaging in social situations that might elicit negative emotions and deploy more attention to positive information. A meta-analysis of memory and attention for emotional stimuli, for example, reflected smaller negativity preferences for older than for younger adults (Murphy and Isaacowitz, 2008). Nevertheless, although older adults have been found to rate the valence of remembered events more positively, this effect appears to reflect a generally positive overall mindset rather than emotion regulation strategies related to personal autobiographic memory (Schryer and Ross, 2012). Findings about emotion experience and the positivity effect in older age are mixed, however. Charles et al. (2010), for instance, found that reduced exposure to daily stressors in older age partially explained age-related reductions in negative affect.

Shallcross et al. (2012) found differences across emotion categories, with lower levels of experienced anxiety and anger, but not sadness. Regarding age differences in the recall of experienced emotions, Grühn et al. (2005) found no aging bias favoring memory of positive material, whereas Röcke et al. (2011) suggested that the correspondence between momentary and retrospective ratings are similar at older and younger ages. Previous studies suggest that life context creates emotional stability in older age; that is, the exposure, not the reactivity, to daily stressors has been found to differ between younger and older people (Sliwinski et al., 2009; Brose et al., 2013).

Taken together, previous studies suggest age differences in emotional lives, but there is no clear consensus about the role of emotional vs. contextual processes leading to emotional positivity in older age. The role of proactive and reactive emotion regulation strategies is a critical factor in understanding the emotional world during aging, whether the positivity effect comes from the lesser experience of negative emotions, or is the experience regulated down by cognitive reappraisal reflected in retrospective emotion ratings. The focus of the current study is to explore whether experienced daily emotions moderate the positivity of retrospective evening ratings—whether the positivity is moderated by the experience and whether the pattern is similar for different emotion categories.

### Personality and retrospective emotion ratings

In addition to age, personality traits could also be expected to interact with emotion recall. There are systematic links between personality and the affective experience, with neuroticism predisposing the experience of more negative, and extraversion more positive emotions (Costa and McCrae, 1992; Watson and Clark, 1992). According to Robinson and Clore (2002), personality-related beliefs can bias reports about retrospective emotion experiences as personality constitutes a source of knowledge that can be used when reporting emotions felt in the past.

More recent studies have found other Big Five personality traits, in addition to neuroticism and extraversion to be also associated with individual differences in emotional processes in daily life (Komulainen et al., 2014). Conscientiousness have found to predict lower levels of negative affect, agreeableness is associated with lower negative and higher positive affect, whereas openness is related to higher stress-reactivity (Komulainen et al., 2014). Neuroticism and extraversion have been found to influence retrospective ratings, with more neurotic people remembering having experienced more negative emotions and more extraverted people more positive emotions (Barrett, 1997).

However, age and valence effects in emotional memory do not change when neuroticism is included as a covariate (Spaniol et al., 2008). Thus, there is no clear consensus about the extent to which personality traits influence the formulation of retrospective emotion ratings, and about whether there are any differences at the time level (the influence of recent vs. long-term emotional memories).

### Daily tiredness and emotional processing

Daily tiredness is a common phenomenon in everyday life, a universal sensation that is considered a natural response to life strain, caused by strenuous activities and emotional stress (Mengshoel, 2010). The life satisfaction and positive affect experienced during the day has been found to be strongly influenced by sleep quality and tiredness, among other variables (Kahneman et al., 2004). Previous studies have suggested that impaired sleep quality leads to low positive affect (Sonnentag et al., 2008; Bower et al., 2010), whereas the association between sleep and mood is bi-directional (Vandekerckhove and Cluydts, 2010). Zohar et al. (2005) found that fatigue resulting from sleep loss, amplified negative emotions as a reaction to an unpleasant event and suppressed positive affect as a response to positive event. Sleep loss and tiredness have been linked to increased emotional lability and deficits in emotion regulation (Dahl and Lewin, 2002; Yoo et al., 2007). In the context of the accessibility model of emotional self-report (Robinson and Clore, 2002), the daily tiredness can operate as a systematic retrospective bias at the episodic memory level, being specific to time and space, and affecting the accessibility to contextual details of event. In addition to accessibility, tiredness can be expected to influence short-term memory about emotion events via mood congruency mechanisms (Rusting and DeHart, 2000) or general arousal level can act as an interference factor (Robinson and Clore, 2002).

Although previous work supports the widespread lay assumption that sleep quality and tiredness appear to be linked to affective functioning, there is need for ambulatory designs for stronger inferences (Bower et al., 2010). Previous studies have also reached to contradictory results, as a study by Peeters et al. (2006) found no relationships between sleep quality and daily affect, whereas Bower et al. (2010) provided evidence that sleep quality is a predictor of positive affect. However, previous studies have also suggested that subjective feeling of fatigue is better predictor of depression than sleep problems (Koffel and Watson, 2009). On the other side, higher levels of trait positive affect as a disposition, not a state, have found to be associated with better overall sleep quality (Ong et al., 2013). Poorer sleep quality impairs individual's ability to use cognitive reappraisal to regulate negative emotions (Mauss et al., 2013). A recent review, Deliens et al. (2014) conclude that exposure to emotional experiences changes sleep-patterns, with emotional disturbances developing after sleep problems.

Taken together, previous studies suggest that sleep plays important role in one's ability to manage emotional information. While, overall, there is more research about illness-related fatigue, emotion studies have so far paid less attention to daily tiredness as a possible influencing factor of the retrospective ratings of experienced emotions. Walker and Harvey (2010) conclude that while mood and tiredness resulting from impaired sleep, are unquestionably linked, the exact nature and outcomes of this relation remains to be determined. Kashani et al. (2012) found that individuals reporting a natural trend for high stress levels reported also a greater daytime sleepiness, tiredness, poorer sleep quality and duration. It is also proposed that personality traits might modulate the link between emotional experiences and sleep quality, whereas the exact nature of this is relation is not clear (Deliens et al., 2014).

The current research includes state-level daily tiredness rated at the end of day and trait-level tiredness across 2 weeks as possible influencing factors in retrospective emotion rating, leading, thus, to the possible enhancement of retrospective ratings of negative emotions and to the possible reduction of positive affect.

### Aims of the current study

Taken together, previous studies have suggested the link between sleep quality and emotion experience, as well as the link between personality traits (i.e., neuroticism and extraversion) and remembering one's emotional experiences. The main aim of the current study was to determine sources of systematic bias in retrospective ratings of the momentary experience of four emotions: fear, sadness, and anger as prototypical negative emotions, and happiness as prototypical positive emotion (Russell and Barrett, 1999; English and Carstensen, 2014), both over a period of 1 day and of 2 weeks. More specifically, it is assumed that retrospective ratings are formulated not only on the basis of experienced emotions, but are also influenced by participant's daily tiredness, age, and personality traits, and that these patterns differ meaningfully across emotion categories. As the purpose of biases in memory for emotions is to enhance one's emotional coping and support coherent self-view (Robinson and Clore, 2002), the systematic biases might differ across discrete emotions.

In summary, we hypothesized that the tiredness, personality traits and age moderate the relationship between momentary emotion ratings and retrospective ratings of 1 day (hypothesis 1) and 2 weeks (hypothesis 2), with meaningful differences across the two time-frames. It was also expected that there is interaction between age and experienced emotion that influences the way in which past feelings are remembered and serves as an emotion regulation strategy (hypothesis 3). As previous studies have suggested Big Five personality traits to have different impact on daily emotional life, the impact of personality traits on the retrospective emotion ratings was expected to differ across the four measured emotion categories (hypothesis 4).

## Materials and methods

### Ethical considerations

The Research Ethics Committee of the University of Tartu approved the study, and all participants provided written informed consent. All the research procedures were conducted in accordance with the Declaration of Helsinki.

### Participants

This study is a part of a larger research project concerning emotion experience in daily life (see also Kööts et al., 2011, 2012). The sample of this project consisted of 110 participants (70 women and 40 men), with ages ranging from 19 to 84 years. All participants were ethnic Estonians and received EEK 520 (about EUR 33) for taking part. The first group of participants (*n* = 55; 42 women and 13 men) was recruited from 2 day centers. The age of participants in this group ranged from 61 to 84 years, with a mean age of 68.2 (*SD* = 5.5). About one-third (36%) of these older respondents had higher education. The second group of participants (*n* = 55; 28 women and 27 men) consisted of undergraduate students from the University of Tartu, and was recruited via advertisements placed in university academic buildings and residence halls. Students came from different faculties of the university and those majoring in psychology were not eligible to participate. The mean age of students was 21.3 years (*SD* = 1.0), ranging from 19 to 23 years.

### Procedure

#### Experience sampling data

The study consisted of 14 days of experience sampling using iESP software (http://seattleweb.intel-research.net/projects/ESM/iESP.html). Participants were signaled randomly 7 times per day during average waking time, to report their current emotions (i.e., up to 99 possible assessments per participant). There were 10,667 measurement trials of momentary emotion across all participants, with an average of 97 measurement trials per participant. The response rate was 82.8%, which is considered to be within normal range for an experience-sampling study (Zelenski and Larsen, 2000). Participants were asked to indicate on a 4-point Likert-type scale (1—not at all to 4—to a large extent), as used in other ESM studies (Mroczek et al., 2003; Gerstorf et al., 2009; Thompson et al., 2011), the extent to which each of the seven basic emotions (anger, happiness, contempt, disgust, fear, sadness, and surprise), as well as six other emotion-related features (disappointed, irritated, in physical pain, sleepy, hungry, and tired) described their current emotional and physiological state as quickly and accurately as possible. Considering the focus of the current paper, four emotions were included in subsequent analyses: happiness (*M* = 2.05, *SD* = 0.62), fear (*M* = 1.12, *SD* = 0.25), and sadness (*M* = 1.34, *SD* = 0.44), and anger (*M* = 1.11, *SD* = 0.22). Momentary tiredness was included as a variable of interest (*M* = 1.83, *SD* = 0.64).

#### Additional measures

For the whole period of 14 days, participants were asked in the evening to recall their emotions for the day. The evening questionnaire consisted of 21 terms about emotional and physiological state, with the instruction to assess the extent to which they had experienced the respective states, averaging these across the day on a 4-point Likert-type scale (1—not at all to 4—to a large extent). The questionnaires were brought back at the end of the study. The four emotions were used for current study: happiness (*M* = 1.24, *SD* = 0.97), fear (*M* = 1.24, *SD* = 0.57), and sadness (*M* = 1.57, *SD* = 0.83), and anger (*M* = 1.29, *SD* = 0.68); and tiredness (*M* = 2.28, *SD* = 0.97).

Participants completed the Estonian version (Allik and Realo, 1997) of the Positive and Negative Affect Schedule (PANAS; Watson et al., 1988), which asks the extent to which they had experienced different emotions during the previous 2 weeks, at the end of the experience sampling period. For current study, the respective items from PANAS reflecting the four emotion terms of happiness (*M* = 2.78, *SD* = 0.80), fear (*M* = 1.45, *SD* = 0.78), and sadness (*M* = 2.15, *SD* = 0.96), and anger (*M* = 1.85, *SD* = 0.86); and tiredness (*M* = 2.46, *SD* = 0.87) were used.

At the beginning of the study, participants also filled in the Estonian version (Kallasmaa et al., 2000) of the Revised NEO Personality Inventory (NEO-PI-R; Costa and McCrae, 1992).

#### Analyses

As a first step, experienced emotions were averaged separately for each emotion category (sadness, fear, happiness, and anger), to produce a mean of all momentary affect ratings across 1 day. There were 1535 mean daily ratings across all participants. Previous studies have suggested mean average momentary assessment to be the best predictor of retrospective ratings of emotions (Röcke et al., 2011).

#### The multilevel regression analysis predicting evening retrospective emotion ratings

A multilevel regression approach was used to explore the moderating role of personality traits and daily tiredness, in addition to experienced momentary emotion, in predicting the retrospective emotion ratings across 1 day and across the 2 weeks. A series of multilevel models was conducted in the Mixed module of IBM SPSS 20.0 with restricted maximum likelihood estimation (REML). The retrospective emotion ratings of Fear_ij_, Sadness_ij_, Happiness_ij_, and Anger_ij_were modeled using a multilevel random intercept model in which the Level 1 random intercept was predicted by mean momentary emotion ratings, personality traits and daily tiredness. As the first step, a no-predictor model was developed to partition the variance in retrospective emotions into within- and between-person components, in order to determine how much of the variance lies between people. Next, a two-level model for analyzing both within- and between-person variability was built. At Level 1, respective retrospective emotion_ij_ represents the emotion rating for participant *j* at in the evening or across the 2 weeks *i* as the outcome variable, where β represents the intercepts, *r* is the respective residual component, ε represents the error term:

##### Level 1 model:

$$Evening\;rating\;of\;fear_{ij},sadness_{ij},anger_{ij}\;or\;happiness_{ij}=\beta_{0j}+r_{ij}+\varepsilon_{ij}.$$

At Level 2, a set of predictors was added to the model. It was proposed that the age, personality traits (neuroticism, extraversion, openness to experience, agreeableness, and conscientiousness) and tiredness might explain differences in retrospective emotion ratings between individuals at the end of the day. The momentary emotions were taken into account, to control the effect of personality traits and tiredness above momentary emotions.

##### Level 2 models:

$$\beta_{0j}\;=\gamma_{00}+\gamma_{01}respective\;mean\;momentary\;emotion_{j}+\mu_{0j}+\varepsilon_{ij},$$

$$\begin{array}{l} \beta_{0j}=\gamma_{00}+\gamma_{01}respective\;mean\;momentary\;emotion_{j}\;\\ \;+\gamma_{02}tiredness\;at\;the\;end\;of\;the\;day_{j}+\gamma_{03}neuroticism_{j}\\ \;+\gamma_{04}extraversion_{j}+\gamma_{05}openness\;to\;experience_{j}\\ \;+\gamma_{06}agreeableness_{j}+\gamma_{07}conscientiousness_{j}+\gamma_{08}age_{j}\\ \;+\gamma_{09}age*mean\;momentary\;emotion_{j}+\mu_{0j}+\varepsilon_{ij}, \end{array}$$

where μ_0__*j*_ *and* ε_*ij*_ refer to random error terms, i.e., the random part of the model.

In addition, as previous studies have suggested age differences in emotion experience, the interaction between age and momentary emotion were taken into account, indicated as age^*^mean momentary emotion.

The model criteria were explored in order to examine the improvement of the null model and the following fixed-effects model. The intraclass correlation (ICC), describing the proportion of between-persons variance to the total variance was also calculated at each level.

#### The linear multiple regression analysis predicting retrospective emotion ratings across the 2 weeks

A linear multiple regression analysis was used to explore the role of personality traits and tiredness in predicting retrospective emotion ratings over the period of 2 weeks. The main purpose of the analysis was to explore whether retrospective ratings of experienced emotions over the 2 weeks are formed on the basis of evening and/or daily emotional experiences, and whether the influence of age, tiredness, and personality traits is similar to evening evaluations. As the data had only between-people variance, a linear multiple regression analysis was used with the emotion experience of the five measured emotions over the 2-week period as the dependent variable and momentary emotion, evening evaluations, age, personality, and daily tiredness as predictor variables.

## Results

First, the correlations between the mean of momentary emotion ratings, mean evening emotion ratings, and 2-week ratings, measuring the experience of the respective emotions across the two study weeks, were explored. Emotion ratings taken at different moments in time were all significantly correlated (Table 1); correlations were moderate, similar to previous studies (Röcke et al., 2011). Thus, the results are in accordance with previous studies, suggesting that people's perceptions of their past emotional experiences are only in part derived from their momentary experiences, and other significant influencing factors can be expected to exist (Barrett, 1997).

**Table 1:** **Pearson correlations between mean momentary emotion ratings, evening ratings, and 2-week ratings of respective emotions**.

**Emotion**	**Two-week and mean momentary emotion**	**Two-week and evening ratings of daily emotions**	**Mean daily and evening ratings**
Happiness	0.38^**^	0.36^**^	0.43^**^
Fear	0.22^**^	0.42^**^	0.30^**^
Sadness	0.34^**^	0.47^**^	0.50^**^
Anger	0.18^**^	0.31^**^	0.40^**^

*N = 1484*.

** *Correlations are significant at the p < 0.01 level (2-tailed)*.

### Retrospective emotion ratings across 1 day

Hypothesis 1 predicted that age, personality traits and tiredness moderate the relationship between momentary emotion ratings and retrospective emotion ratings of happiness, fear, sadness, and anger across the period of 1 day.

The results of the null model, Level 1 random intercept model, and Level 2 fixed-effects models are presented comparatively in Tables 2–5, separately for each of the four emotions.

**Table 2:** **The evening ratings of fear—results of the mixed models analysis of the null model and fixed effects random intercept model**.

**Model**	**Predictors**	***−2RLL***	**Estimates of fixed effects**					**Estimates of covariance parameters**				
			***EST***	***SE***	***df***	***F***	***Sig*.**	***ICC***	***Wald Z* across subjects**	***Wald Z* within subjects**	***R^2^* change Level 1**	***R^2^* change Level 2**
The null model	Intercept	2207	1.24	0.03	109.15	1418.66	0.00	32.14%	6.39^**^	26.43^**^		
The fixed effects models	Intercept	2145	0.69	0.07	994.07	87.88	0.00	28.47%	6.19^**^	26.41^**^	3.19%	18.62%
	Mean momentary fear		0.49	0.06	1479.20	68.64	0.00					
	Intercept	2194	0.48	0.22	320.24	4.82	0.03	23.66%	5.70^**^	26.38^**^	0.39%	22.43%
	Mean momentary fear		0.80	0.13	1480.70	38.97	0.00					
	Age		0.00	0.00	492.30	0.03	0.86					
	Tiredness		0.04	0.02	1478.37	5.01	0.03					
	Age^*^daily emotion		−0.00	0.00	1415.72	6.42	0.01					
	N		0.00	0.00	100.89	0.05	0.83					
	E		−0.00	0.00	102.75	0.01	0.91					
	O		−0.00	0.00	100.80	0.10	0.75					
	A		0.00	0.00	99.58	0.17	0.68					
	C		0.00	0.00	101.80	0.05	0.83					

** *p < 0.001; N, neuroticism; E, extraversion; O, openness to experience; A, agreeableness; C, conscientiousness; −2RLL refers to −2 Restricted Log Likelihood*.

**Table 3:** **The evening ratings of sadness—results of the mixed models analysis of the null model and fixed effects random intercept model**.

**Model**	**Predictors**	***−2RLL***	**Estimates of fixed effects**					**Estimates of covariance parameters**				
			***EST***	***SE***	***df***	***F***	***Sig*.**	***ICC***	***Wald Z* across subjects**	***Wald Z* within subjects**	***R^2^* change Level 1**	***R^2^* change Level 2**
The null model	Intercept	3151	1.58	0.05	109.12	852.18	0.00	42.42%	6.72^**^	26.44^**^		
The fixed effects models	Intercept	2997	0.74	0.08	573.28	96.22	0.00	31.46%	6.16^**^	26.37^**^	7.33%	42.26%
	Mean momentary sadness		0.63	0.05	1466.88	181.58	0.00					
	Intercept	2940	−0.07	0.21	186.93	0.10	0.75	15.92%	4.96^**^	26.34^**^	3.54%	7.23%
	Mean momentary sadness		1.01	0.09	1220.47	134.23	0.00					
	Age		0.00	0.00	351.81	1.27	0.26					
	Tiredness		0.14	0.02	1402.41	48.49	0.00					
	Age^*^daily emotion		−0.01	0.00	1216.66	27.95	0.00					
	N		0.00	0.00	97.83	1.47	0.23					
	E		−0.00	0.00	99.23	1.16	0.28					
	O		0.00	0.00	96.72	1.58	0.21					
	A		0.00	0.00	96.19	1.59	0.21					
	C		−0.00	0.00	97.22	0.05	0.83					

** *p < 0.001; N, neuroticism; E, extraversion; O, openness to experience; A, agreeableness; C, conscientiousness; −2RLL refers to −2 Restricted Log Likelihood*.

**Table 4:** **The evening ratings of happiness—results of the mixed models analysis of the null model and fixed effects random intercept model**.

**Model**	**Predictors**	***−2RLL***	**Estimates of fixed effects**					**Estimates of covariance parameters**				
			***EST***	***SE***	***df***	***F***	***Sig*.**	***ICC***	***Wald Z* across subjects**	***Wald Z* within subjects**	***R^2^* change Level 1**	***R^2^* change Level 2**
The null model	Intercept	3732	2.43	0.06	109.24	1722.76	0.00	35.54%	6.52^**^	26.42^**^		
The fixed effects models	Intercept	3562	1.31	0.09	683.31	202.90	0.00	30.06%	6.27^**^	26.40^**^	10.43%	30.17%
	Mean momentary happiness		0.54	0.04	1495.66	205.58	0.00					
	Intercept	3565	1.35	0.31	174.30	19.05	0.00	27.04%	5.87^**^	26.36^**^	3.60%	16.87%
	Mean momentary happiness		0.71	0.07	1493.89	92.34	0.00					
	Age		0.01	0.00	304.66	1.64	0.20					
	Tiredness		−0.14	0.03	1486.85	31.78	0.00					
	Age^*^daily emotion		−0.01	0.00	1462.99	8.62	0.00					
	N		−0.00	0.00	100.12	4.54	0.04					
	E		0.01	0.00	102.09	19.31	0.00					
	O		−0.00	0.00	100.17	0.57	0.45					
	A		−0.00	0.00	98.95	1.42	0.24					
	C		−0.00	0.00	100.18	0.04	0.85					

** *p < 0.001; N, neuroticism; E, extraversion; O, openness to experience; A, agreeableness; C, conscientiousness; −2RLL refers to −2 Restricted Log Likelihood*.

**Table 5:** **The evening ratings of anger—results of the mixed models analysis of the null model and fixed effects random intercept model**.

**Model**	**Predictors**	***−2RLL***	**Estimates of fixed effects**					**Estimates of covariance parameters**				
			***EST***	***SE***	***df***	***F***	***Sig*.**	***ICC***	***Wald Z* across subjects**	***Wald Z* within subjects**	***R^2^* change Level 1**	***R^2^* change Level 2**
The null model	Intercept	2973	1.29	0.03	109.22	1708.09	0.00	17.37%	5.47^**^	26.43^**^		
The fixed effects models	Intercept	2760	0.05	0.09	1279.38	0.27	0.60	13.24%	4.95^**^	26.41^**^	7.33%	42.26%
	Mean momentary anger		1.13	0.07	1493.09	235.00	0.00					
	Intercept	2765	−0.65	0.21	512.41	9.07	0.00	7.96%	3.84^**^	26.40^**^	2.49%	44.78%
	Mean momentary anger		1.89	0.14	1464.14	173.82	0.00					
	Age		0.02	0.00	878.16	21.87	0.00					
	Tiredness		0.06	0.02	1150.66	9.66	0.00					
	Age^*^daily emotion		−0.02	0.00	1399.89	40.79	0.00					
	N		0.00	0.00	104.21	1.64	0.20					
	E		−0.00	0.00	108.37	3.03	0.08					
	O		0.00	0.00	104.49	0.55	0.46					
	A		−0.00	0.00	103.41	0.89	0.35					
	C		0.00	0.00	105.22	0.12	0.73					

** *p < 0.001; N, neuroticism; E, extraversion; O, openness to experience; A, agreeableness; C, conscientiousness; -2RLL refers to -2 Restricted Log Likelihood*.

First, the effects of mean momentary emotion ratings were explored. The experienced mean momentary emotions had significant effect on retrospective emotion ratings for all emotions (fear: γ = 0.483, *p* < 0.01; sadness γ = 0.628, *p* < 0.01; happiness γ = 0.543, *p* < 0.01; anger: γ = 1.130, *p* < 0.01). The reductions in variance estimates (*R*^2^) suggest that the mean of momentary emotion ratings of the respective emotion accounts for about 3 (fear) to 10 (happiness) percent of the within-people variability, and for about 18 (fear) to 42 (anger) percent of the between-persons variability in evening emotion ratings. Thus, a significant portion of the variability in means of evening ratings across people can be attributed to differences in the daily emotion experience. However, the Wald Z test suggests that, even after controlling for the mean of momentary emotion ratings within-people, a statistically significant amount of variation in outcomes still remains both within- and between people for all four emotions (*p* > 0.05).

Next, the effect of Level 2 fixed predictors was explored. Individuals who reported more tiredness at the evening, tended to enhance the retrospective ratings of negative emotions (the effect of tiredness for fear: γ = 0.036, *p* < 0.01; for sadness γ = 0.141, *p* < 0.01; for anger: γ = 0.058, *p* < 0.01) and reduced retrospective rating of happiness γ = −0.142, *p* < 0.01, compared to reported mean momentary emotion.

The Level 2 models suggest that tiredness impacts the evening ratings of all measured emotions, magnifying negative emotions and reducing happiness. Retrospective evening ratings of happiness were associated with neuroticism (γ = −0.004, *p* < 0.05) and extraversion (γ = 0.010, *p* < 0.01). There were no significant effects for evening retrospective ratings for other emotions (*p* > 0.05). The effects of age were included in model both as single predictor, but also in interaction with reported mean momentary emotions (hypothesis 3). Older adults have found to report less negative emotions and more positive emotions in general (Carstensen et al., 2011), however, it is possible that in situations where negative emotions are experienced, the retrospective ratings of older adults may be different. The age effect on retrospective emotion ratings was significant only for anger (γ = 0.018, *p* < 0.01), suggesting that older adults tended to retrospectively enhance the experienced anger compared to mean momentary assessments. There was a significant interaction between mean momentary emotion and age in predicting retrospective emotion ratings for sadness (γ = −0.010, *p* < 0.01), for fear (γ = −0.006, *p* < 0.05), for anger (γ = −0.020, *p* < 0.01), and for happiness (γ = −0.005, *p* < 0.01), supporting the hypothesis 3. Thus, the more older adults experienced both negative and positive emotions, the more it was biased in evening retrospective ratings as less intense. Adding fixed predictors significantly improved the model, there was observable R^2−^change at between-people level for all measured emotions. The hypothesis 1 was partly supported as daily tiredness and age were found to moderate the relationship between momentary emotion ratings and retrospective ratings across 1 day. Surprisingly, personality traits had no significant influence on emotional experience across 1 day, except for happiness.

### Retrospective ratings across 2 weeks

Next, the retrospective ratings over the 2-week period were assessed in order to test the hypothesis 2.

The results of the multiple regression analysis (Table 6) suggest tiredness, personality traits, and age to be important predictors of emotion evaluation over the 2-week period, supporting the hypothesis 2. Hypothesis 4 predicted that the influence of personality traits on retrospective emotion ratings differs across the measured emotion categories. Nearly all personality traits are significantly related to trait-level emotion ratings, with the trait neuroticism being important predictor for all emotion categories (*p* < 0.05). The retrospective ratings of fear are influenced by higher neuroticism (β = 0.12, *p* < 0.001) and lower extraversion (β = −0.21, *p* < 0.001). The retrospective ratings of sadness are predicted by personality traits of neuroticism (β = 0.28, *p* < 0.001), extraversion (β = −0.13, *p* < 0.001), openness to experience (β = 0.11, *p* < 0.05), and conscientiousness (β = −0.06, *p* < 0.05). The retrospective ratings of happiness are predicted by all big five personality traits: neuroticism (β = −0.31, *p* < 0.001), extraversion (β = 0.13, *p* < 0.001), openness to experience (β = 0.24, *p* < 0.001), agreeableness (β = −0.08, *p* < 0.05), and conscientiousness (β = −0.10, *p* < 0.05). The retrospective ratings of anger are predicted by neuroticism (β = 0.15, *p* < 0.001), openness to experience (β = 0.15, *p* < 0.001), and conscientiousness (β = −0.13, *p* < 0.001). There are also differences across emotion categories, with the assessment of experienced anger and sadness across the 2 weeks depending more on evening ratings than momentary emotions—if the emotion is strong enough to be presented in evening ratings, then it also affects the emotion experience across the 2 weeks.

**Table 6:** **Multiple regression analysis of emotion ratings across the 2 weeks—mean of respective momentary emotion ratings, respective evening emotion ratings, personality traits, tiredness, and age as predictors**.

**Two-week emotion rating**	**Model summary**			**Predictors (Standardized coefficient beta)**								
	***R***	***R^2^***	***SE***	**N**	**E**	**O**	**A**	**C**	**Age**	**Mean of momentary emotion ratings**	**Evening ratings**	**Tiredness**
Fear	0.54	0.29	0.66	0.12^**^	−0.21^**^	0.04	0.06	0.04	−0.24^**^	0.13^**^	0.31^**^	0.10^**^
Sadness	0.70	0.45	0.71	0.28^**^	−0.13^**^	0.11^*^	0.03	−0.06^*^	−0.27^**^	0.09^**^	0.22^**^	0.05^**^
Happiness	0.55	0.30	0.75	−0.31^**^	0.13^**^	0.24^**^	0.08^*^	−0.10^*^	0.22^**^	0.26^**^	0.15^**^	0.08^**^
Anger	0.59	0.34	0.70	0.15^**^	0.05	0.15^**^	0.03	−0.13^**^	−0.27^**^	0.03	0.18^**^	0.04

* p < 0.05;

** *p < 0.001; N, neuroticism; E, extraversion; O, openness to experience; A, agreeableness; C, conscientiousness*.

## Discussion

The current study extends previous research by exploring the effect of momentary emotions, daily tiredness, and personality on the retrospective ratings of four emotions across two age-groups across different time frames.

Previous studies have demonstrated age-related positivity effect in emotion experience (Carstensen et al., 2011). This emotional positivity can be also reflected in retrospective emotion ratings. Consistent with the third hypothesis, the association of age and retrospective emotion ratings is moderated by the felt momentary emotions. The stronger were the momentary emotions, the stronger was the age effect, suggesting that retrospective reduction of the intensity of experienced emotions may serve as one of reactive emotion regulation mechanisms behind the positivity effect.

### The impact of personality on retrospective emotion ratings

Consistent to our fourth hypothesis, the associations between personality traits and retrospective emotion ratings differed both across emotions and time frames. The retrospective ratings of happiness are linked to personality traits of extraversion and neuroticism already in the evening ratings of the past day. At longer time frame, all Big Five personality traits have impact on emotional memories about happy feelings. Memories about experienced fear are influenced only by neuroticism and extraversion. Retrospective ratings of sadness are strongly related to neuroticism, but are also predicted by extraversion, openness and conscientiousness. Retrospective ratings of anger, however, are predicted by neuroticism, openness, and conscientiousness. However, a significant part of variability in all retrospective emotion ratings was still left unexplained by the model; this might be due to the influence of emotions experienced at the moment of recall that were not measured in the current study (i.e., situational beliefs or self-esteem as proposed by Robinson and Clore, 2002). Our study extended the results of recent study on the association between daily affect and personality traits (Komulainen et al., 2014) that showed the role of all Big Five personality traits in emotion processes. Similarly, conscientiousness was associated with lower affect levels, whereas openness to experience predicted retrospective enhancement of experienced emotions.

Taken together, current study shows the role of personality traits in affecting memories of experienced emotions, with all Big Five personality traits having a significant role in formulating retrospective emotion ratings.

### Tiredness and retrospective emotion ratings

Tiredness is a very common condition; it can be physical, psychological, or social. In any case, we can assume it to have an impact on one's emotional world, whereas experienced emotions can be both a cause and a consequence of tiredness. Previous studies have rather focused on sleep quantity and quality and related affect regulation problems, our findings contribute to the current knowledge of the association between tiredness and retrospective assessments of experienced emotions. There is significant influence of daily tiredness on retrospective emotion ratings across 1 day. The feeling of tiredness at the end of the day makes one to enhance the experienced negative emotions of fear, sadness and anger. In addition, the experienced happiness is seen less intense when rated at the state of evening tiredness. This result is in accordance with previous studies and supports the theorized link between the experience of tiredness and anxiety (Jiang et al., 2003; Kahneman et al., 2004). Alternatively, it has been suggested that pre-sleep period at the evening is the first quiet time during the day available to review the day's events and one's own behavior, that may lead to more precise self-perception (e.g., Schmidt and Van der Linden, 2009). In addition, tiredness also influences retrospective ratings across 2 weeks. In general, the reported subjective feeling of tiredness is linked to affective functioning, similar to the results reported by sleep studies (Bower et al., 2010). It is suggested that at the end of the day, people have a quiet moment in order to analyze the experienced events and behavior. Findings provide further evidence for the accessibility model of emotional self-report (Robinson and Clore, 2002), suggesting that, although retrospective ratings are based on momentary experience, daily tiredness and personality traits systematically influence the way in which past feelings are seen. Our study confirms that tiredness systematically influences the memories about experienced emotions, and may, thus, lead to more negative view of life.

### Age and retrospective ratings

Age-related differences in both emotion experience and emotional memory are well documented by previous research. The current study confirms age patterns found in previous studies, suggesting that the emotional world becomes more stable as people age (Carstensen et al., 2011). One important contribution of the current study is that age effects in retrospective ratings were found to be moderated by the experience of respective momentary emotions. The current study provides empirical support for the SOC-ER framework by Urry and Gross (2010), suggesting that the positivity effect in older adults is achieved by more effective situation selection, which brings about less negative and more positive emotions. Age-related shift of retrospective emotion rating toward more positive look is more reflected at trait-level emotion experience, and less at state level. If, however, the situation selection approach does not work and older people experience negative emotions, then the retrospective ratings are used as means for emotion regulation. The memories of experienced emotions are regulated to less intense, compared to momentary ratings. Whereas, this retrospective reassessment takes place also in the case of experienced happiness. Previous studies also show that for older people the intentional suppression of unwanted memories is more difficult (Anderson et al., 2011). Perhaps this is the wisdom that comes with experience: the use of proactive vs. reactive emotion regulation strategies. However, when momentary emotions are experienced, there is a stronger impact on emotional life for older than for younger people.

### The remembering of past emotions

The current study also investigated the remembering past emotions, and the factors that can be associated with the distortion of these memories. The results indicate that even when momentary emotions, age effects, personality traits, and daily tiredness are taken into account, a significant amount of variability is still left to be explained. This might reflect the influence of post-event emotions and reappraisals that shape the emotional memory. This finding is in line with previous research suggesting that a tendency exists to achieve coping in the present by reconstructing the past (Levine and Safer, 2002). Also, in the context of the accessibility model of emotional self-report (Robinson and Clore, 2002), current research suggests that personality processes are involved rather in the long-term modification of emotional memory than during that of 1 day, operating based on semantic knowledge and one's beliefs about emotions. Also, age has more influence at trait-level, suggesting that older people tend to assess experienced emotions retrospectively into positive direction. Interestingly, tiredness can also be regarded as systematic biasing factor that interferes emotion reports both at online level, tied to specific situations, as well as at the level of semantic memory over longer periods of time.

### Implications and limitations

Identifying the systematic biases in retrospective emotion ratings that are produced by trait-level variables like age and personality traits can foster the better understanding of individual differences in daily emotional life. Our findings contribute to the current knowledge of the associations between momentary and retrospective emotion ratings, further confirming the existence of recall bias in retrospective assessment of emotions. From methodological point of view, previous studies (e.g., Sato and Kawahara, 2011) have used evening emotion ratings vs. 2-week ratings of mood. The results of our study suggest also the evening emotion ratings to be subject of both state- and trait level biases. The results further suggest that when using retrospective emotion ratings, both in clinical or research setting, one should also take into account the current psychological state (e.g., tiredness) and also the trait-level influence factors (e.g., age and personality traits). Previous studies have shown the influence of neuroticism and extraversion on remembering the past emotions (Barrett, 1997). Our study makes specific contribution in showing that also openness to experience, conscientiousness and agreeableness as traits shape the memories about emotional experiences.

Several potential limitations of the study can be noted. The most important limitation of our study is that the study sample included only two age-groups, and therefore the results cannot directly be generalized to whole population. We only used emotion ratings averaged across 1 day, future studies could also look for peak-end rule in memories of experienced emotions. In addition, the evening questionnaires were paper-and-pen questionnaires, leaving the possibility of back-filling. Future research could be directed to advancing the understanding of other biases in memory for emotions at different points of time (e.g., different coping strategies used in different situations, etc.), and could include also middle-aged people to shed light into emotional changes across the entire lifespan.

## Conclusions

Taken together, the results of the current study showed that systematic biases in retrospective emotion ratings across 1 day come from tiredness and age, whereas retrospective emotion ratings across the 2 weeks are also influenced by personality traits. Considering tiredness, personality traits, and the age effect provides new insight into individual differences in retrospective emotion ratings in the context of accessibility model of emotional self-report (Robinson and Clore, 2002). The current study replicated the previous findings of a positivity effect in emotional experience with age and extended the prior literature by showing that age-related positivity in emotional life can be partially attributed to successful use of retrospective reassessment as an emotion regulation strategy. Personality traits, however, appeared to influence a more long-term view of past emotional experience, compared to the 1-day perspective. Future research could be directed to advancing the understanding of other biases in memory for emotions, and could include also middle-aged people to shed light into emotional changes across the entire lifespan.

### Conflict of interest statement

The authors declare that the research was conducted in the absence of any commercial or financial relationships that could be construed as a potential conflict of interest. The reviewer and handling Editor declared their shared affiliation, and the handling Editor states that the process nevertheless met the standards of a fair and objective review.
